# American Society for Enhanced Recovery (ASER) and Perioperative Quality Initiative (POQI) Joint Consensus Statement on Optimal Analgesia within an Enhanced Recovery Pathway for Colorectal Surgery: Part 2—From PACU to the Transition Home

**DOI:** 10.1186/s13741-017-0063-6

**Published:** 2017-04-13

**Authors:** Michael J. Scott, Matthew D. McEvoy, Debra B. Gordon, Stuart A. Grant, Julie K. M. Thacker, Christopher L. Wu, Tong J. Gan, Monty G. Mythen, Andrew D. Shaw, Timothy E. Miller, For the Perioperative Quality Initiative (POQI) I Workgroup, Michael Scott, Matthew McEvoy, Debra Gordon, Stuart Grant, Julie Thacker, Christopher Wu, Tong Gan, Monty Mythen, Andrew Shaw, Timothy Miller

**Affiliations:** 1 Department of Anesthesiology, Virginia Commonwealth University Health System, 1200 East Broad Street, P.O. Box 980695, Richmond, Virginia 23298-0695 USA; 2 University College London, London, UK; 3 CIPHER (Center for Innovation in Perioperative Health, Education, and Research), Vanderbilt University Medical Center, TN, USA; 4 Department of Anesthesiology, Vanderbilt University School of Medicine, 2301VUH,, Nashville, TN 37232 USA; 5 Department of Anesthesiology & Pain Medicine, Harborview Integrated Pain Care Program, University of Washington, Seattle, WA USA; 6 Department of Anesthesiology, Medical Student Education, Division of Regional Division, Duke University Medical Center, Durham, UK; 7 Department of Surgery, Division of Advanced Oncologic and GI Surgery, Duke University Medical Center, Durham, UK; 8 Department of Anesthesiology/Critical Care Medicine, The Johns Hopkins University School of Medicine, Baltimore, MD USA; 9 Department of Anesthesiology, Stony Brook University School of Medicine, Stony Brook, USA; 10 University College London Hospitals National Institute of Health Research Biomedical Research Centre, London, UK; 11 Department of Anesthesiology, Vanderbilt University, TN, USA; 12 Division of General, Vascular and Transplant Anesthesia, Duke University Medical Center, Durham, UK

**Keywords:** Enhanced recovery pathway, Colorectal surgery, Analgesia, Optimal analgesia, Pain management, Multimodal, Non-opioid adjuncts, Postoperative, Post-discharge, Outcomes, Quality

## Abstract

**Background:**

Within an enhanced recovery pathway (ERP), the approach to treating pain should be multifaceted and the goal should be to deliver “optimal analgesia”, which we define in this paper as a technique that optimizes patient comfort and facilitates functional recovery with the fewest medication side effects.

**Methods:**

With input from a multidisciplinary, international group of experts and through a structured review of the literature and use of a modified Delphi method, we achieved consensus surrounding the topic of optimal analgesia in the perioperative period for colorectal surgery patients.

**Discussion:**

As a part of the first Perioperative Quality Improvement (POQI) workgroup meeting, we sought to develop a consensus document describing a comprehensive, yet rational and practical, approach for developing an evidence-based plan for achieving optimal analgesia, specifically for a colorectal surgery within an ERP. The goal was twofold: (a) that application of this process would lead to improved patient outcomes and (b) that investigation of the questions raised would identify knowledge gaps to aid the direction for research into analgesia within ERPs in the years to come. This document details the evidence for a wide range of analgesic components, with particular focus on care in the post-anesthesia care unit, general care ward, and transition to home after discharge. The preoperative and operative consensus statement for analgesia was covered in Part 1 of this paper. The overall conclusion is that the combination of analgesic techniques employed in the perioperative period is not important as long as it is effective in delivering the goal of “optimal analgesia” as set forth in this document.

## Introduction

As noted in Part 1 (McEvoy MD et al. 2016), pain after major abdominal surgery is severe and is a major component of the stress response if not adequately treated, and a structured, multicomponent plan is needed throughout all phases of care (Schricker & Lattermann 2015). This Part 2 consensus document focuses on all phases of postoperative care ranging from the post-anesthesia care unit (PACU) to the surgical ward to the transition to home and post-discharge period. We present a structured approach to achieving optimal analgesia as well as an algorithm for rescue analgesia for patients in whom optimal analgesia has not been achieved or who have breakthrough pain. The document ends with a discussion of appropriate outcomes related to analgesia as well as future directions for research.

## Methods

The methodology and process followed for this Perioperative Quality Initiative (POQI) I subgroup (optimal perioperative analgesia) are presented in Part 1 (McEvoy MD et al. 2016). The overall POQI process and members of the POQI 1 work group are described in the editorial that accompanies this series (Miller TE et al. 2016).

## Results

Considering the postoperative phase of care related to achieving optimal analgesia, our group arrived at the following list of questions as being those most pertinent to and all-encompassing of the topic of optimal analgesia as a component of an enhanced recovery pathway (ERP) for colorectal surgery:

1. How can optimal analgesia be achieved while minimizing opioid use during all postoperative phases for colorectal surgery?
2. What are the effective strategies for treating patients who need rescue analgesia?
3. How can analgesia be measured in a clinically meaningful way?
4. What are the research gaps that exist in regards to perioperative analgesia in colorectal surgery in ERPs? What are the proper research designs and methods by which to answer these questions?

Q1: How can optimal analgesia be achieved whilst minimizing opioid use during all postoperative phases for colorectal surgery?

Statement: Optimal analgesia after colorectal is achieved through a planned multimodal analgesia approach minimizing opioid use during all phases of perioperative care.

**Fig. 1:**
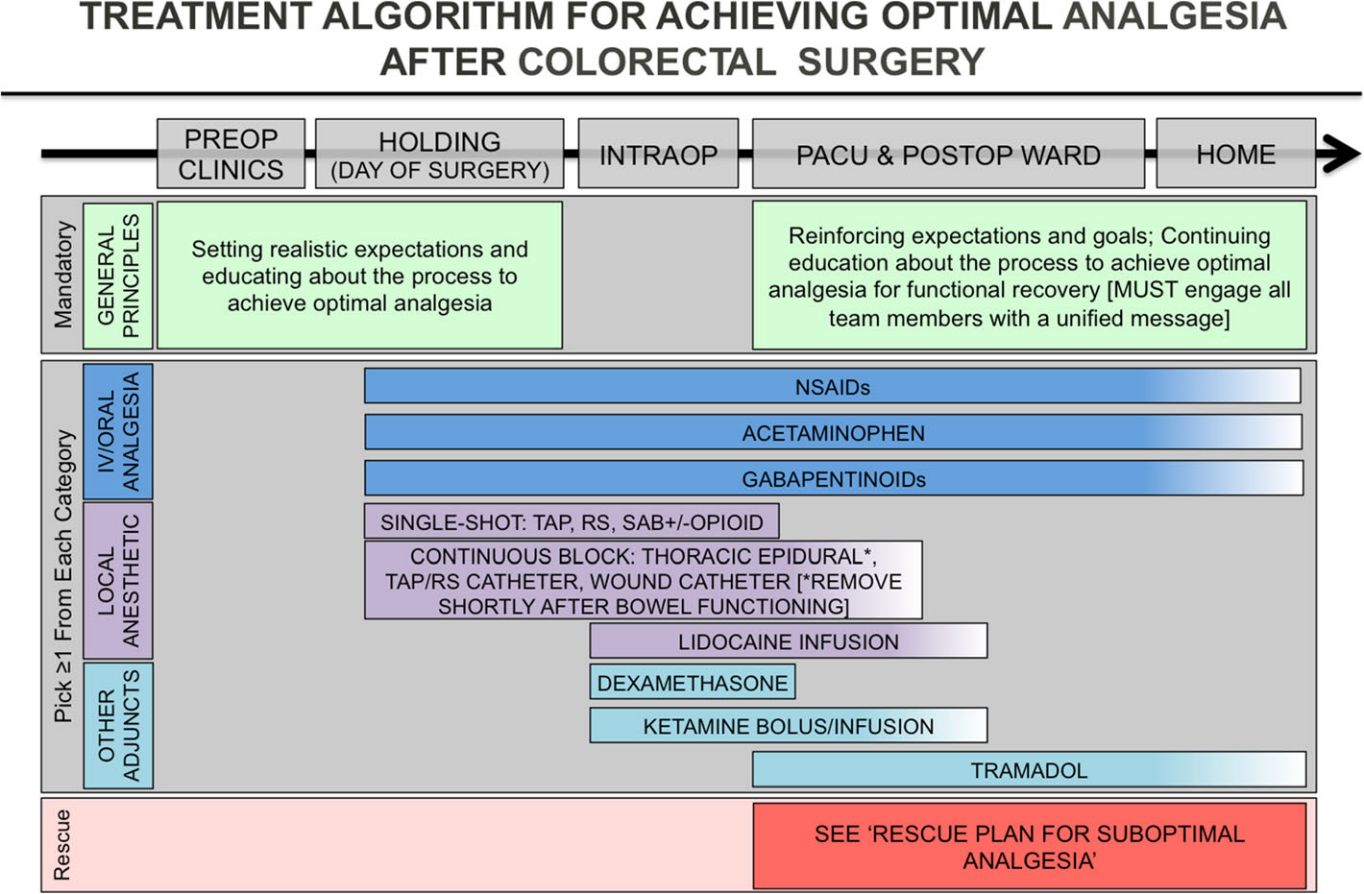
This figure illustrates suggested components of a multimodal approach to pain management in an ERP for colorectal surgery. Of note, the plan should be comprehensive, encompassing all phases of perioperative care from preoperative to post-discharge

In order to deliver optimal analgesia, a well-structured and planned multimodal approach should be constructed that spans from the preoperative period into the post-discharge recovery phase (see Fig. 1).

## Postoperative-PACU to Ward

### Neuraxial

#### Spinal anesthesia

Spinal anesthesia is placed preoperatively but still needs to be considered: First, as the spinal wears off and there is a transition in analgesia required and second, if long-acting opioids have been added then vigilance regarding postoperative respiratory depression is required.

#### Thoracic epidural anesthesia

Following open colonic surgery, a continuous epidural infusion placed at an appropriate spinal level to provide coverage of the wound should be used. Ideally, the epidural infusion can be commenced before surgery and continued intraoperatively and postoperatively. The pain of laparoscopic colectomy reduces more quickly than open surgery due to the smaller abdominal incisions making the benefits for a continuous epidural less clear (Chung et al. 2007). Laparoscopic colectomy can still be as painful as open colectomy particularly in the first 24 h (Maartense et al. 2004). Patient indications include significant preexisting pulmonary impairment or chronic opioids use with tolerance. Thoracic epidural infusions employing a combination of local anesthetic and opioid provide the best analgesia in colorectal surgery but addition of opioids to an epidural solution can slow bowel motility (Jorgensen et al. 2000). Local anesthetic or opioid alone can also be infused where indicated.

### Paravertebral blocks, peripheral catheters, or TAP blocks

Transversus abdominis plane (TAP) blocks and paravertebral blocks are commonly placed preoperatively and have been discussed in the Part 1 of the paper. TAP blocks appear to provide longer analgesia than surgical infiltration analgesia and in laparoscopic surgery are non-inferior to epidurals (Park et al. 2015; Niraj et al. 2014). Various approaches have been tried successfully in different surgeries. Paravertebral blocks, along with TAP blocks, TAP catheters, and wound infiltration have been employed in many studies. Due to design bias, unblinding and small sample size generalization and recommendations are limited but these blocks should be considered postoperatively in patients where other techniques have failed.

### Intravenous medications

#### Lidocaine

In the recovery room and postoperatively, a continuous lidocaine infusion at a rate of approximately 1–1.5 mg/kg/hr IV for up to 48 h postoperatively has been shown to be beneficial (McEvoy et al. 2016; Wongyingsinn et al. 2011; Ventham et al. 2015). The optimal dosing and duration of lidocaine is still not known, and the side effects are directly related to the serum lidocaine level (Khan et al. 2016a)

#### N-methyl-D-aspartate antagonists

Ketamine infusions can be employed as a supplemental or rescue analgesia technique in patients who have failed initial management in the postoperative period. Low dose ketamine infusions can be used on a general floor and do not require additional nursing interventions or personnel (Jouguelet-Lacoste et al. 2015). When compared with opioids alone, ketamine has been shown in meta-analysis and systematic reviews to demonstrate improved pain control and reduced PONV without increases in adverse side effects (Jouguelet-Lacoste et al. 2015; Wang et al. 2016; Ding et al. 2014). Ketamine should be considered as an adjunct for those experiencing pain not adequately controlled by other schedule non-opioid medications and is particularly useful in patients on long-term opioid medication. Low-dose ketamine (2 mcg/kg/min IV) after major abdominal surgery has been shown to reduce postoperative opioid requirements (Laskowski et al. 2011; Sami Mebazaa et al. 2008; Zakine et al. 2008).

#### Acetaminophen and Non-Steroidal Anti-Inflammatory Drugs (NSAIDs)

Acetaminophen and NSAIDs have been reported as the backbone of multimodal analgesia in the postoperative period of ERPs. There is evidence that they can reduce the need for opioids by 15–50% (Elia et al. 2005; Jibril et al. 2015; O’Neal 2013; Smith 2011; Toms et al. 2008). It is reasonable, barring any contraindications, to provide scheduled oral or IV doses of acetaminophen and NSAIDs in the postoperative period.

### Opioids: oral, IV, and PCA—when and for whom?

**Fig. 2:**
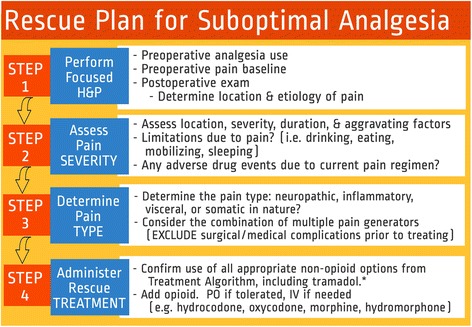
This figure illustrates a structured approach as a rescue plan for a patient experiencing suboptimal pain control. Except in extreme cases, this step-by-step process should lead to appropriate management that continues the principles being employed with the goal of delivering optimal analgesia

Although one of the main analgesic goals for ERPs is minimization of opioid use, one must recognize that opioids are still an important analgesic option; however, their role is less central (i.e., used more as an “as needed basis,” pro re nata (PRN)) when compared to traditional care where opioids where the main analgesic options for postoperative pain management. For opioid-naïve patients who are not yet tolerating oral intake, patients ideally would have one of the main local anesthetic-based options and multimodal analgesia before utilizing short-acting IV opioids for rescue analgesia (see Fig. 2). After these, opioid-naïve patients are tolerating oral intake, then short-acting oral opioids can be added for breakthrough pain *after* a scheduled regimen of non-opioid analgesics such as gabapentin, acetaminophen, NSAIDs, and other multimodal analgesic agents (McEvoy et al. 2016; Smith 2011; Schmidt et al. 2013).

There are surprisingly few RCTs examining IV patient-controlled analgesia (PCA) vs. no IV PCA for patients undergoing laparoscopic surgery (including colon resection). One retrospective analysis in 297 patients undergoing laparoscopic surgery for colorectal cancer compared a group who received conventional opioid-based PCA postoperatively to a non-PCA group who received intravenous anti-inflammatory drugs as necessary. There was no difference in pain scores or use of rescue analgesia on POD 1–5. As such, the authors suggest that IV PCA may not be necessary in selected patients those who underwent minimal invasive surgery for colorectal cancer (Choi et al. 2015). Another recent publication reported improved functional recovery with an ERP for CRS patients in which the use of IV PCA opioids went from 94% in historical controls (*N* = 179) to <5% after ERP implementation (*N* = 365) and overall opioid use was reduced by ~80% with no change in pain scores (McEvoy et al. 2016). Thus, although some chronic pain patients will benefit from a PCA, the assumption that IV opioids via PCA should be a standard part of postoperative recovery is not necessarily valid and should be reconsidered. For specific considerations with respect to the chronic pain patient, see Part 1.

#### Opioid receptor antagonists

Ileus is one of the most frequent issues increasing hospital length of stay in patients undergoing colorectal surgery with associated increase in healthcare costs. The cause of ileus is multifactorial but opioids have a significant impact (Hubner et al. 2015). Novel agents that are μ-opioid receptor antagonist of μ-opioid receptors within the gastrointestinal tract are thought to block the gastrointestinal effects of opioids without blocking the analgesic efficacy, thus reducing the likelihood of opioid-induced constipation or ileus. In a meta-analysis of three randomized controlled trials examining the efficacy if 12 mg of alvimopan (vs. placebo) in patients undergoing abdominal surgery in an accelerated recovery program found that alvimopan significantly reduced time to the hospital discharge and gastrointestinal recovery (Vaughan-Shaw et al. 2012). A meta-analysis of patients undergoing laparoscopic surgery (although not necessarily in an accelerated recovery program) found a reduction in postoperative ileus development when patients were given alvimopan compared to placebo; however, the other factors contributing to ileus were not controlled in these studies (Nguyen et al. 2015).

### Tramadol

Tramadol has been classified both as an opioid and as a non-opioid based upon its unique mechanism of action (Vazzana et al. 2015). There are no studies examining tramadol as a substitute for opioids in the postoperative period in colorectal surgery patients. One review suggested that tramadol may reduce the opioid requirement but this did not have an impact on clinical outcomes (Martinez et al. 2015). The authors recommend the use of tramadol as one postoperative adjunct and in a rescue analgesia treatment algorithm prior to using traditional opioids (see Fig. 2).

## Postoperative—transition from ward to home

### Transition process in-hospital

No specific data exists concerning the exact process by which transitions should occur in between analgesic modalities during the hospital stay. Pain intensity decreases over the first few postoperative days depending on both the degree of visceral injury and size and position of abdominal wall incisions. A major factor in achieving adequate analgesia while minimizing opioid use is the establishment of early oral intake in the immediate postoperative period and commencement of oral multimodal analgesia.

From a practical perspective, it is reasonable to remove the TEA catheter when the patient is passing flatus. One study reported using a postoperative lidocaine infusion for 24 h in all CRS patients, either immediately for those who received truncal blocks or after TEA catheter removal for those receiving an epidural, which typically delineated laparoscopic versus open cases, respectively, (McEvoy et al. 2016). Furthermore, a number of other studies have reported beneficial effects on length of stay, opioid consumption, nausea and vomiting, and gastrointestinal function with the use of lidocaine infusions for 24–28 h postoperatively (Wongyingsinn et al. 2011; Ventham et al. 2015). Additionally, an ultra-low-dose ketamine infusion (e.g., 2 mcg/kg/min) may be of benefit (Ding et al. 2014; Laskowski et al. 2011; Sami Mebazaa et al. 2008) Maximum utilization of non-opioid medications should be used throughout the postoperative period and continued after a TEA catheter is removed and/or IV infusions are discontinued. After this point, oral opioids should be used for rescue analgesia, but this should be accomplished only with PRN dosing.

### At time of hospital discharge

#### Medications

Few studies are available to guide the clinician with regard to the precise most optimal dosing, start and stop time for different perioperative interventions. The incidence of severe pain decreases over each day from surgery through the first 1–2 weeks after surgery. Several meta-analyses have indicated that certain medications given preoperatively are associated with a decrease in postoperative pain and opioid consumption. These medications include celecoxib, (Khan et al. 2016b), acetaminophen (De Oliveira et al. 2015; Doleman et al. 2015a), gabapentin (Doleman et al. 2015b; Seib and Paul 2006), and pregabalin (Mishriky et al. 2015). There is also evidence that continuing NSAIDs, acetaminophen, and gabapentinoids postoperatively as part of a multimodal analgesia pathway is associated with improved outcomes (McEvoy et al. 2016; Schmidt et al. 2013; Larson et al. 2014). However, given the method by which these studies were completed (quasi-experimental pre-post design involving a care bundle), it is not possible to know the exact effect and necessary duration for continuing these medications. For practical purposes, an example of the specific medications and dosages used in one successful ERP for CRS are included in Table 1 (McEvoy et al. 2016). However, it should be noted that there are a variety of successful approaches concerning the specific analgesic components for applying the principle of opioid avoidance presented in this consensus statement (Larson et al. 2014; Miller et al. 2014; Thiele et al. 2015).

**Table 1:** An example of components of an ERP for colorectal surgery patients utilizing maximodal non-opioid analgesia^a^

Perioperative period	Components	Adjustments/Notes
Preoperative	Gabapentin: 300–600 mg PO >1 hour before OR time	- Reduce to 300 mg PO in patients >65y - Consider not giving or reducing to 100 mg PO in patients >75y - Consider dose reduction in patients with OSA
	Acetaminophen: 1000 mg PO >1 hour before OR time	- Reduce to 650 mg PO if <70kg - Don’t use if h/o significant liver disease
	Bilateral TAP Blocks ± rectus sheath blocks OR thoracic epidural catheter	- TAP - ropiv 0.25% + dex 4mg (25–30mL/side) - Rectus sheath - ropiv 0.25% + dex 2 mg (10–12mL/side) [add rectus sheath blocks for if any portion of incision [e.g. periumbilical handport] or large ports above umbilicus] - Thoracic epidural used for midline incision extending from above T8 to below umbilicus [use during intraoperative period]
Intraoperative	No induction opioids; minimize opioid use during anesthetic	- Volatile agent or propofol anesthetic in addition to ketamine - Esmolol for heart rate control
	Ketamine: 0.5 mg/kg with induction bolus *plus* 5mcg/kg/min until fascia closure.	- Consider reducing bolus (0.25mg/kg) or not using bolus in elderly patients >65 years of age.
	Lidocaine: 1.5 mg/kg bolus with induction then 2mg/min drip from induction to case end	- Contraindications: Unstable heart disease, recent MI, heart block, heart Failure, electrolyte disturbances, liver disease, seizure disorder, current anti-arrhythmic therapy [e.g. amiodarone, sotalol]
	Ketorolac: 30 mg IV at fascia closure	- Reduce to 15 mg IV if >65y, CrCl < 30, or patient weight <50kg. - Consider avoiding for h/o renal dysfunction or GI bleed
	Methadone: Consider methadone 10–20 mg IV with induction for patients with chronic opiate use; may consider higher doses based on home opioid regimen.	- If opioids required, consider methadone on emergence or in PACU (5 mg IV boluses) q5–10 min prior to using other opioids.
Postoperative	Gabapentin: 300-600 mg PO TID starting DOS until discharge	- Use lower dose for >65y or if patient having sedation/dizziness - Post-discharge: final inpatient dose PO TID × 7 days, then half dose PO TID × 7 days [2 week post-op course total]
	Acetaminophen: 1000 mg PO Q8hr starting DOS until discharge	- Reduce to 650 mg PO Q6h if <70kg - Post-discharge: 500–1000mg PO Q8h × 3 days and then PRN
	Lidocaine	Continued from PACU or after thoracic epidural catheter removed Order for PACU to continue 24h: 1 mg/min IV if <70 kg; 1.5 mg/min IV if 70–100 kg; 2 mg/min IV >100 kg. Contraindications as above
	Ketorolac: 30mg IV q6h × 3 days	- Reduce to 15 mg IV Q6h in patients >65y, CrCl < 30, or weight <50kg - Hold if evidence of acute kidney injury
	Opioids: as needed (PRN)	Example: Oxycodone 5mg PO Q4 PRN pain >4/10; consider opioid PCA or PRN bolus for breakthrough pain, but not a standard order. - Post-discharge: short course of short-acting opioid (e.g. oxycodone 5mg q6h PRN × 3days) unless chronic pain/opioid use concerns to address.
	Thoracic Epidural	If used, continue with local anesthetic (e.g. bupivacaine 0.1%) +/- opioid if needed for denser quality block (e.g. hydromorphone 10mcg/mL)

^a^It should be noted that this is one example of a successful ERP for CRS, but there are many approaches concerning the specifics of medications and doses. *Ropiv* ropivicaine, *dex* dexamethasone, *mL* milliliter, *mg* milligram, *TAP* transversus abdominis plane, *PACU* post anesthesia care unit, *PCA* patient-controlled analgesia

#### Education

Patients and families should be reassured that some discomfort, particularly with movement, is normal (Alawadi et al. 2016). Encouragement to use non-pharmacologic and non-opioid interventions as frontline management is essential to successful opioid-sparing approaches (Sugai et al. 2013). The impact of continued use of acetaminophen, NSAIDs and gabapentinoids beyond the acute hospital recovery phase is unknown, but some may benefit from several weeks of low risk, non-opioid analgesia (Schmidt et al. 2013). Assure a uniform process to inform the patient and family which provider will be responsible for managing postoperative pain and provide instructions on the planned taper of postoperative analgesics, including a timeline for return to preoperative or lower opioid dosing for those on chronic opioids (Rose et al. 2016). Adding new prescriptions of opioids, benzodiazepines, sedative-hypnotics, anxiolytics or central nervous system depressants at the time of transition out of the hospital should be avoided. If opioids are continued at discharge, patients and families should be counseled about the risks of co-administration with alcohol and other central nervous system depressants, as well as the dangers of prescription opioid diversion. Teams should instruct the patient and family on the importance of secure storage of their medications and prompt disposal of controlled substances either through a Drug Enforcement Agency (DEA) approved take-back program or Food and Drug Administration (FDA) guideline for safe disposal of medicine (Rose et al. 2016). The agreed preoperative plan to taper off opioids added for surgery as surgical healing takes place should be followed. The goal is always the shortest duration and lowest effective dose whenever using opioids. It may be appropriate to discharge patients on acetaminophen, NSAIDs, and/or gabapentinoids only, or with only a very limited supply of short-acting opioids (e.g., 2–3 days)—even if they were taking opioids preoperatively.^1^

Q2: What are effective strategies for troubleshooting patients who need rescue analgesia?

Statement: Use a stepwise approach including assessment of potential causes, maximizing non-opioid approaches and if needed using a brief course of short-acting opioid.

Conventional opioid rescue treatment commonly termed “breakthrough” dosing is not the first approach that should be employed when optimal analgesia is not achieved, as it can rapidly negate effects of opioid-sparing regimens (Cooney 2016). When pain is poorly controlled, it is important to establish the etiology and consider other treatable causes. In addition to incisional pain, common causes of pain following CRS may include bowel distention from flatus, diaphragmatic irritation referred to the shoulder, or muscle wall soreness caused by stretch and cramping. Pain that does not respond to usual treatment may signal a complication or otherwise treatable cause. A structured approach for “rescue” analgesia should be in place, such as described in Fig. 2. Additionally, because pain is always an emotional experience attention to the patient experience (e.g., understanding of goals, coping strategies and fears) is important in all rescue steps.

Q3: How can analgesia be measured in a clinically-meaningful way?

Statement: Measurement of analgesia after colorectal surgery should occur through a system that accounts for patient experience and overall function.

A more detailed framework for comprehensive measurement of quality of care relevant to enhanced recovery programs (ERPs) in elective colorectal surgery is detailed elsewhere (Moonesinghe SR et al. 2016). Here, we focus on outcome measures related to the treatment of pain. Measurement of optimal analgesia is complex and dependent on the purpose (e.g., clinical point-of-care decision making, quality improvement or research). Outcome domains recommended for assessment of single-dose acute pain analgesic trials include pain intensity, pain relief, adverse effects and physical function such as the ability to ambulate without assistance, and other clinically relevant recovery measures (Cooper et al. 2016). In the clinical setting, pain intensity ratings documented at variable time periods in the patient record are often tempting as an outcome measure for enhanced recovery teams, however, may not accurately reflect the patient experience and are void of physical function. Median and range values of patient report of pain severity using a 0–10 numerical rating scale at specified time periods yielding the Sum of Pain Intensity Differences (SPID) or Total Pain Relief Score (TOTPAR) at 24 and 48 h on movement and rest are the commonly used measures in research studies, but data capture is labor intensive and not practical for most ERP teams. Also, a systematic review of movement-evoked pain (e.g., deep breathing, ambulation) versus pain at rest in postsurgical clinical trials concluded that the assessment of movement-evoked pain is poorly defined and has been neglected (Srikandarajah and Gilron 2011).

A QI dataset for optimal analgesia would ideally include a measure of DREAMS (Drinking, Eating, Analgesia, Mobilizing, Sleeping); however, to date, there is no specific validated research tool. Administration of a validated health-related quality of life questionnaire such as the EuroQoL (EQ5D) or the Medical Outcomes Short-Form Health Survey (SF-36), before surgery and at various time points postoperatively is ideal but may not be readily achievable (Brazier et al. 1993; Patt & Mauerhan 2005). Researchers and clinicians are increasingly recognizing the importance of using assessments that reflect experience in the “real world”. Two multidimensional patient-reported outcome instruments designed for use in the first day after surgery have been validated including the revised American Pain Society Patient Outcome Questionnaire (APS-POQ-R) and the International Pain Outcome (IPO) Questionnaire (Gordon et al. 2010; Rothaug et al. 2013). Both capture domains of pain and pain relief, impact of pain on emotions and physical function, treatment side effects, and perceptions of care and can be recommended for use in quality improvement studies. The IPO is of particular value because it provided comparative benchmarking data as part of the only international acute postoperative pain registry, Pain-Out (http://pain-out.eu).

An important aspect of the patient experience is satisfaction. Patient satisfaction is a notoriously complicated outcome measure that has long been paradoxical in nature in pain management studies. In 2008, the Centers for Medicare and Medicaid started publicly reporting the results of the Hospital Consumer Assessment of Healthcare Providers and Systems (HCAHPS) survey. The HCAHPS survey is the first standardized national survey that publicly reports patients’ perceptions of their hospital care. The survey evaluates the patient’s experience of their hospital stay and their overall rating and satisfaction of the hospital. One domain of the survey is pain management. The pursuit of high HCAHPS performance has been blamed for pressure on medical providers to honor patient requests for unnecessary and in some cases potentially harmful amounts of opioids. Decoupling pain management HCAHPS scores from hospital payment scoring calculations is one of several recent Health and Human Services actions designed to reduce opioid use. Nevertheless, the HCAHPS pain composite scores for various subgroups of patients, such as those on enhanced recovery pathways, can serve as an important driving force for resources and change.

Concerning other validated measures, it appears that PROMIS instruments faithfully capture information in respondents’ natural environments and reflect the participants’ real-life symptom, but the number of components for future work should be limited in order to reduce patient burden and the analytic workload while delivering results (Stone et al. 2015; Bingener et al. 2015). In the absence of a Health-Related Quality of Life (HRQoL) instrument specifically validated for the postoperative pain setting, Taylor and colleagues used two generic questionnaires, the 12-item Short-Form health survey (SF-12) and EQ-5D in general surgery and orthopedic procedure patients to measure impact of pain on quality of life on postoperative day seven (Taylor et al. 2013). Multivariate regression analyses showed that irrespective of confounding factors (e.g., age, gender, and preoperative HRQoL) patients with severe postoperative pain experience important reductions in both physical and mental well-being domains of their HRQoL. Long-term outcomes impacted by perioperative analgesia include persistent postsurgical pain (PPP), quality of life, and cancer recurrence. An observational study in Europe on patients with general surgery and orthopedic procedures reported that a 10% increase in the percentage of time spent in severe pain in the first day after operation was associated with a 30% increase of PPP at 12 months, indicating the amount of time spent in severe pain was more significant than a single-pain intensity (Fletcher et al. 2015). Specific evaluation for colorectal surgery on the consequences of PPP for quality of life and function has not yet been validated (Kehlet and Jorgensen 2016).

In summary, the outcomes of analgesic technique are diverse in nature, and clinical consequences vary from unpleasant symptoms to mortality (Lee et al. 2015). Traditional outcome measures used in acute pain clinical trials, such as opioid consumption, worst or averaged pain intensity ratings, and length of stay appear to be less than ideal because they are either poorly defined or have little demonstrable clinical value. Rare harm outcomes are nearly absent in the literature in part because they are extraordinarily difficult to measure (Gordon et al. 2016). Teasing out the impact of analgesic strategies among a multifactorial enhanced recovery pathway on important recovery issues may be impossible but an ERP should include analgesia measures that account for patient experience, function, and quality of life.

Q4: What are the research gaps that exist in regards to perioperative analgesia in colorectal surgery?

Statement: Controversy exists surrounding the effects of analgesia management on short and long-term outcomes in colorectal surgery.

There are numerous research gaps that exist in regards to achieving optimal perioperative analgesia in CRS ERPs. Very few individual randomized controlled trials and almost no meta-analysis have examined perioperative interventions concurrently for both CRS patients and ERPs. Future studies in this domain need to investigate the optimal dosing, timing, and combinations of perioperative interventions in CRS ERPs throughout the entire perioperative period, with a focus not only on pain scores and opioid consumption but also on a structured approach to also assess the effect on functional recovery and adverse effect from analgesics. Finally, if standardized national and international data registries become available, then insight into real-world efficacy of specific analgesic components or combinations could be obtained from a regression analysis in a large cohort with excellent compliance from ERPs employing different analgesic strategies.

## Summary and future directions

Delivering optimal analgesia is a key component of enhanced recovery pathways for colorectal surgery. There are many ways to achieve analgesia for patients undergoing CRS. The technique of choice will depend on which surgical procedure is performed and by which approach (laparoscopic, robotic assisted or open). Patient factors including preferences and availability of experienced providers, training, and equipment within a hospital will also determine which technique can be utilized. A multifaceted approach to achieving optimal analgesia is necessary to restore postoperative function as rapidly as possible while minimizing the common side effects of opioid-based analgesia. Further research is needed into identifying better ways of bridging analgesia when stopping the major analgesic choice and steeping down to oral multimodal analgesia. The effect of analgesic modalities on long-term outcomes (particularly cancer) needs to be studied to guide the future direction of analgesic modalities.

## Abbreviations

APS-POQ-R: American Pain Society Patient Outcome Questionnaire
DEA: Drug Enforcement Agency
DREAMS: Drinking, Eating, Analgesia, Mobilizing, Sleeping
EQ-5D: EuroQoL
ERP: Enhanced recovery pathway
FDA: Food and Drug Administration
HCAHPS: Hospital Consumer Assessment of Healthcare Providers and Systems
HRQoL: Health-Related Quality of Life
IPO: International Pain Outcome
IV: Intravenous
NSAIDs: Non-steroidal anti-inflammatory agents
PACU: Post-anesthesia care unit
PCA: Patient-controlled analgesia
POQI: Perioperative quality improvement
PRN: As needed/pro re nata
PROMIS: Patient-Reported Outcome Measurement Information System
SF-12: 12-item Short-Form health survey
SPID: Sum of Pain Intensity Differences
TAP: Transversus abdominis plane
TOTPAR: Total Pain Relief Score

## Footnotes

1 The WA Agency for Medical Directors 2015 Opioid Guideline (last accessed March 24, 2017). 
http://www.agencymeddirectors.wa.gov/Files/2015AMDGOpioidGuideline.pdf
