# Correction to: American Society for Enhanced Recovery (ASER) and Perioperative Quality Initiative (POQI) Joint Consensus Statement on Optimal Analgesia within an Enhanced Recovery Pathway for Colorectal Surgery: Part 2—From PACU to the Transition Home

**DOI:** 10.1186/s13741-018-0086-7

**Published:** 2018-04-10

**Authors:** Michael J. Scott, Matthew D. McEvoy, Debra B. Gordon, Stuart A. Grant, Julie K. M. Thacker, Christopher L. Wu, Tong J. Gan, Monty G. Mythen, Andrew D. Shaw, Timothy E. Miller, For the Perioperative Quality Initiative (POQI) I Workgroup, Timothy E. Miller, Andrew D. Shaw, Michael G. Mythen, Tong J. Gan, Matthew D. McEvoy, Michael J. Scott, Deborah Gordon, Stuart Grant, Julie K. M. Thacker, Christopher L. Wu, Robert H. Thiele, Karthik Raghunathan, C. S. Brudney, Dileep N. Lobo, Daniel Martin, Anthony Senagore, Stefan D. Holubar, Traci Hedrick, John Kellum, Ruchir Gupta, Mark Hamilton, S. Ramani Moonesinghe, Mike P. W. Grocott, Elliott Bennett-Guerrero, Thomas J. Hopkins, Roberto Bergamaschi, Stuart McCluskey

**Affiliations:** 1 Department of Anesthesiology, Virginia Commonwealth University Health System, 1200 East Broad Street, P.O. Box 980695, Richmond, Virginia 23298-0695 USA; 2 University College London, London, UK; 3 CIPHER (Center for Innovation in Perioperative Health, Education, and Research), Vanderbilt University Medical Center, Nashville, TN USA; 4 Department of Anesthesiology, Vanderbilt University School of Medicine, 2301VUH, Nashville, TN 37232 USA; 5 Department of Anesthesiology & Pain Medicine, Harborview Integrated Pain Care Program, University of Washington, Seattle, WA USA; 6 Department of Anesthesiology, Medical Student Education, Division of Regional Division, Duke University Medical Center, Durham, UK; 7 Department of Surgery, Division of Advanced Oncologic and GI Surgery, Duke University Medical Center, Durham, UK; 8 Department of Anesthesiology/Critical Care Medicine, The Johns Hopkins University School of Medicine, Baltimore, MD USA; 9 Department of Anesthesiology, Stony Brook University School of Medicine, Stony Brook, USA; 10 University College London Hospitals National Institute of Health Research Biomedical Research Centre, London, UK; 11 Department of Anesthesiology, Vanderbilt University, TN, USA; 12 Division of General, Vascular and Transplant Anesthesia, Duke University Medical Center, Durham, UK

## Correction

After publication of this article (Scott et al. 2017), it was noticed that the HTML version contained technical errors regarding the collaborator information of the Perioperative Quality Initiative (POQI) I Workgroup. The Declarations section was missing the acknowledgements for the group. The correct information can be found in this correction.
